# Use of VISIA-CR Generation 5 in Aesthetic Skin Assessment: A Single-Case Review

**DOI:** 10.1093/asjof/ojag034

**Published:** 2026-02-13

**Authors:** Carolyn Kim, Bhavana Thota, Jennifer Barillas, John E Hoopman, Jeffrey M Kenkel

## Abstract

Traditional evaluations of nonsurgical aesthetic treatments often rely on clinical photography and patient-reported outcomes, which are subjective and may overlook subtle or subsurface changes. Advanced imaging technologies, such as the VISIA-CR system, have increased objectivity; however, earlier generations provided limited parameters and lacked integrated analytics. The aim of this review was to illustrate the enhanced capabilities of the VISIA-CR Generation 5 system (VISIA-CR 5) and explore its clinical relevance through a single-case example. A 49-year-old female with Fitzpatrick skin phototype III underwent a combined treatment with intense pulsed light, a fractionated 1927 nm laser, and a dual-wavelength 1470/2940 nm laser. Standardized VISIA-CR 5 imaging was performed at baseline and 3 months. The system captured multispectral images and quantified 8 skin features (visible spots, brown spots, red spots, ultraviolet (UV) spots, texture, pores, porphyrins, and wrinkles) using expanded multiparameter analytics. The VISIA-CR 5 provided multidimensional assessment beyond conventional visual evaluation by quantifying feature intensity, background skin tone, and relative contrast. Although the mean intensity of visible, brown, and UV spots appeared darker at 3 months, contrast-based analysis indicated improved skin tone uniformity. The system also detected reduced fractional area alongside increased count for visible and UV spots, suggesting fragmentation of larger spots rather than proliferation, a level of detail unattainable with traditional methods. This case demonstrates that the VISIA-CR 5 can provide detailed imaging and integrated analytics that support comprehensive, objective evaluation of aesthetic treatment outcomes. Its expanded parameters facilitate detection of subtle, clinically relevant changes and highlight its potential utility in clinical research and practice.

**Level of Evidence:** 4 (Therapeutic)

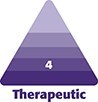

Historically, evaluations of nonsurgical, office-based aesthetic treatments have relied on subjective tools, such as clinical photography, patient-reported outcomes, and blinded observer rating scales.^1-4^ Although these methods provide valuable insights, they lack standardized objective criteria. Furthermore, they depend on visual inspection and often fail to capture subsurface changes. To address these limitations, advanced imaging technologies have emerged to deliver objective, quantifiable, reproducible measurements of skin health and appearance.^5^

One commonly used system in clinical research is the VISIA-CR system (Canfield Scientific, Parsippany, NJ). This facial imaging device captures multispectral images under various lighting conditions to assess 8 key features: visible spots, brown spots, red spots, ultraviolet (UV) spots, texture, pores, porphyrins, and wrinkles.^6-8^ By using different lighting environments, the system can highlight pigmentation, vascular patterns, and textural changes that may not be apparent to the naked eye.^9^ The precision, accuracy, and validity of this imaging system have been well documented in previous studies.^6,10,11^

The latest version, VISIA-CR Generation 5 (VISIA-CR 5), introduces several improvements. These include light-emitting diode flash panels for faster image acquisition, expanded metrics per analyzable feature, and integrated analytical capabilities that eliminate the need for separate software. Collectively, these enhancements allow for more comprehensive and efficient evaluation of skin characteristics, allowing clinicians to detect subtle changes that older systems may have overlooked. Despite these advancements, no published studies have yet provided an in-depth description of the upgraded system's capabilities or its implications for clinical research and practice. To address this gap, we present a patient who received a single combined treatment of intense pulsed light (IPL), a fractionated 1927 nm nonablative laser, and a dual-wavelength 1470/2940 nm laser. Through this case, we aim to illustrate the capabilities of the VISIA-CR 5 system, review its expanded data outputs, and explore their clinical relevance in aesthetic medicine.

## Methods

### Image Acquisition

**Figure 1.**
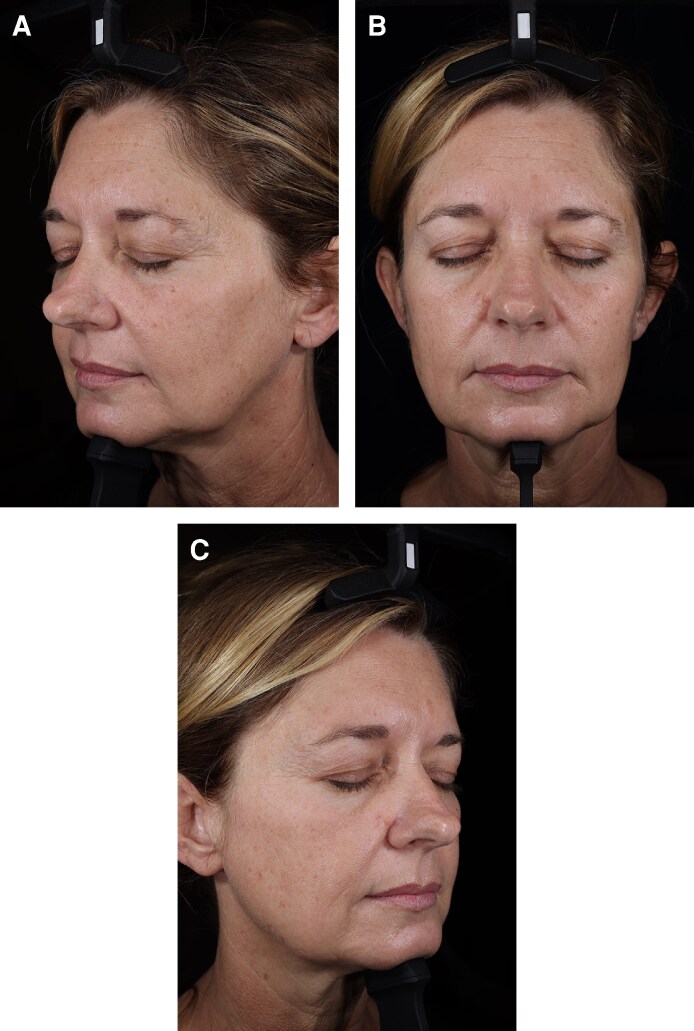
Standardized patient positioning during image acquisition using the VISIA-CR 5 system to capture (A) left oblique, (B) frontal, and (C) right oblique views.

This study was a single-case review conducted in accordance with IRB-approved protocols. Written consent was obtained, permitting the use and analysis of the patient's imaging and data. Facial images were captured using the VISIA-CR 5 camera system (Canfield Scientific, Parsippany, NJ), which provides both qualitative and quantitative assessments across the 8 aforementioned skin features. Prior to imaging, the patient removed all skincare and cosmetic products and acclimated to ambient temperature and humidity conditions for 15 min. The patient was then carefully positioned in the modular head support and instructed to maintain a neutral, nonsmiling facial expression with eyes gently closed. Standardized photographs were acquired at 3 views: a central view (directly facing the camera) and 2 angled views (at 45° to the right and left) at baseline and 3 months posttreatment (Figure 1).

### Data Definitions

**Figure 2.**
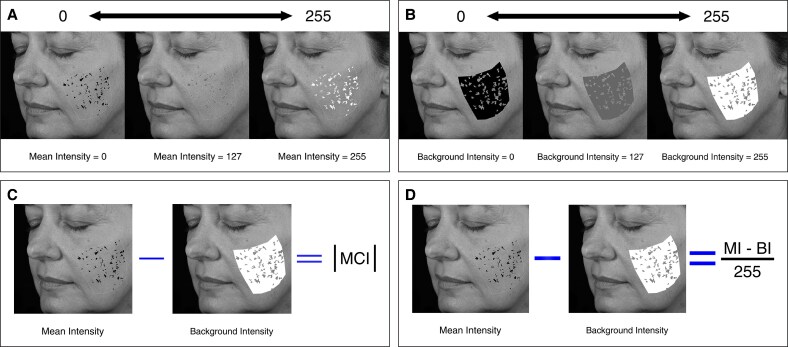
Simulated patient photograph showing the calculations for intensity-related parameters provided by the VISIA-CR 5 imaging system: (A) mean intensity (MI), (B) background intensity (BI), (C) mean contrast intensity (MCI), and (D) intensity.

Mean intensity measured the average intensity of detected features on a scale from 0 to 255, with lower values corresponding to darker features (Figure 2A). Background intensity measured the average intensity within the selected masked region of interest, excluding detected features, and shared the same scale from 0 to 255 (Figure 2B). Mean contrast intensity depicted the difference between mean intensity and background intensity, indicating the relative brightness or darkness of a feature compared with the surrounding skin tone (Figure 2C). Finally, intensity provided similar information as mean contrast intensity but as a normalized value ranging from −1 to +1 (Figure 2D).

**Figure 3.**
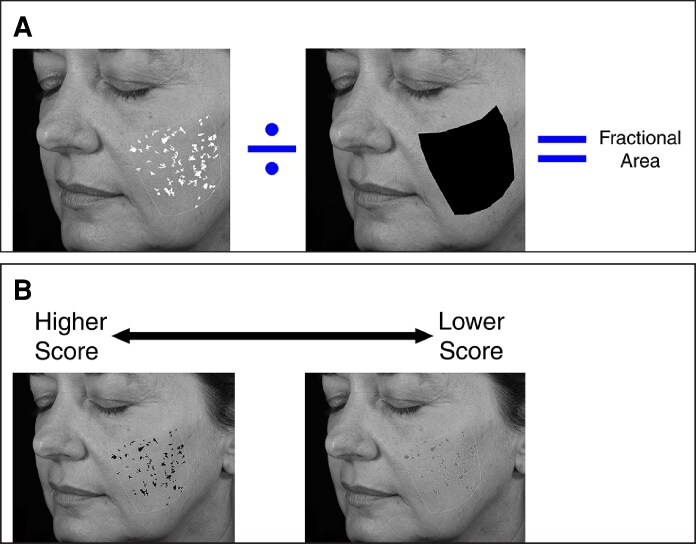
Simulated patient photograph showing the calculations for feature (A) fractional area and (B) score provided by the VISIA-CR 5 imaging system.

Additional parameters included fractional area, calculated as the proportion of the feature area relative to the total masked region (Figure 3A), and count, which referred to the number of individual features detected within the region of interest. Score was a normalized measure of feature severity ranging from 0 to 1, where lower scores indicated less detection (Figure 3B). For all of the spot features (visible, brown, red, and UV spots), the score incorporated both fractional area and intensity, whereas for all other features, it was derived solely from fractional area. Wrinkle-specific parameters included mean thickness, measured as the width at 90° from the long axis along the wrinkle's midpoint, and total area, which was further categorized into size ranges from <0.15, 0.15 to 0.21, 0.21 to 0.28, 0.28 to 0.34, 0.34 to 0.41, 0.41 to 0.47, to >0.47 mm^2^.

### Statistical Analysis

All parameter values were generated directly from the built-in VISIA-CR 5 analytical software. For the left and right facial views, percent change from baseline was calculated using the system-reported measurements for each view. For the central view, the VISIA-CR 5 system provided separate outputs for the forehead and nose; therefore, these values were combined by summing the forehead and nose measurements at baseline and at follow-up. Percent change was then computed from the aggregated baseline and follow-up totals to generate a single percent-change value for the center view.

## Results

The patient was a 49-year-old Caucasian, non-Hispanic female with Fitzpatrick skin phototype III who underwent a single combined treatment consisting of IPL, a fractionated 1927 nm nonablative laser, and a dual-wavelength 1470/2940 nm laser.

### Left View

**Figure 4.**
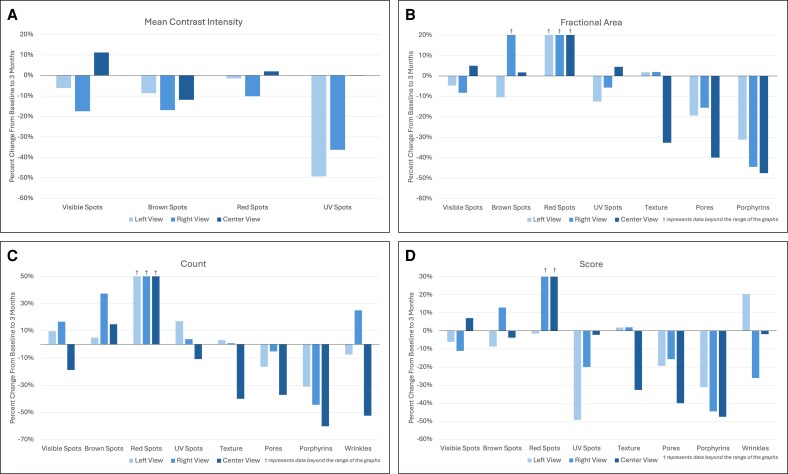
Percent change in (A) mean contrast intensity, (B) fractional area, (C) count, and (D) score of skin features at 3 months using the VISIA-CR 5 imaging system.

**Figure 5.**
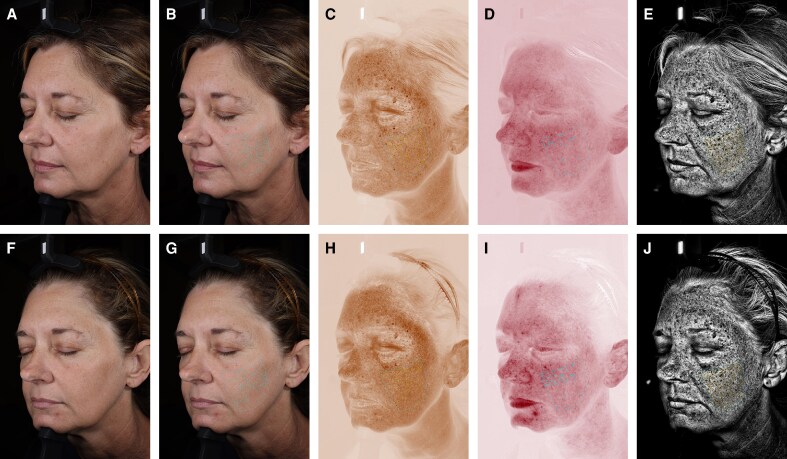
Left oblique view showing pigmentation skin changes in a 49-year-old female at (A-E) baseline and (F-J) 3 months following a single combined treatment using intense pulsed light, a fractionated 1927 nm laser, and a 1470/2940 nm laser. (A, F) Standard lighting, (B, G) standard lighting for visible spot analysis, (C, H) cross-polarized lighting for brown spot analysis, (D, I) cross-polarized lighting for red spot analysis, (E, J) ultraviolet lighting for ultraviolet spot analysis.

For pigmentation changes at 3 months posttreatment, the left view demonstrated a decrease in mean contrast intensity for all spot features, with the greatest reduction seen in UV spots (−49.29%; Figures 4, 5). Fractional area showed mixed results, decreasing for visible (−4.68%), brown (−10.45%), and UV spots (−12.52%) but increasing substantially for red spots (+129.38%). Feature count increased for all spots, including visible (+9.62%), brown (+4.92%), red (+146.67%), and UV spots (+17.02%).

For topographical skin changes, the fractional area of texture minimally increased at 3 months (+1.81%), whereas pores and porphyrins decreased by −19.30% and −31.14%, respectively. Count demonstrated a similar pattern, with a slight increase in texture (+3.07%) but a decrease in pores (−16.39%), porphyrins (−31.07%), and wrinkles (−7.41%). Mean wrinkle thickness (+4.70%) and total wrinkle area (+20.42%) both increased at follow-up. Further subgroup analysis revealed increases in area of all wrinkle sizes except for the 0.15 to 0.21 mm^2^ range, which decreased by −17.97%.

### Right View

The right view showed similar trends to those observed on the left view, with mean contrast intensity decreasing for all pigmented spots. Fractional area decreased for visible (−8.25%) and UV spots (−5.73%) but increased for brown (+31.77%) and red spots (+273.40%). Feature count increased for all spot features, with red spots again showing the largest change (+146.67%).

**Figure 6.**
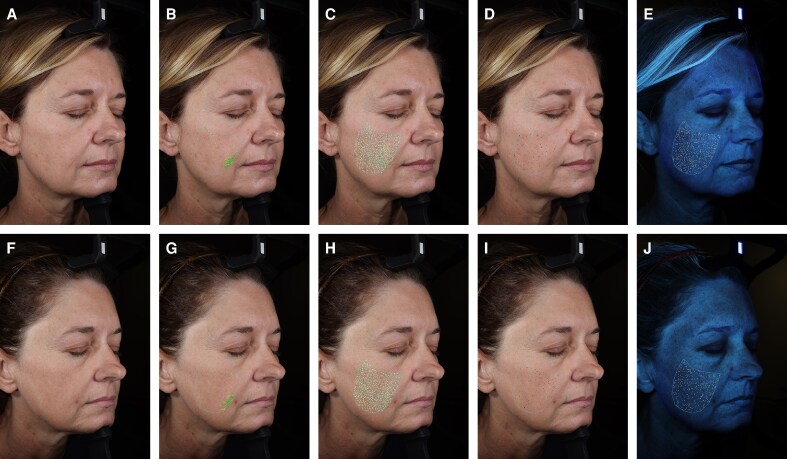
Right oblique view showing topographical skin changes in a 49-year-old female at (A-E) baseline and (F-J) 3 months following a single combined treatment using intense pulsed light, a fractionated 1927 nm laser, and a 1470/2940 nm laser. (A, F) Standard lighting, (B, G) standard lighting for wrinkle analysis, (C, H) standard lighting for texture analysis, (D, I) standard lighting for pore analysis, (E, J) ultraviolet lighting for porphyrin analysis.

Fractional area and count both minimally increased for texture but decreased for pores and porphyrins on the right view (Figure 6). For wrinkles, the count increased by 25.00%, whereas mean thickness (−7.99%) and total wrinkle area (−26.03%) decreased. When analyzed by size distribution, wrinkle area decreased in most size categories but slightly increased in the <0.15 mm^2^ (+9.90%) and >0.47 mm^2^ (+1.34%) ranges.

### Center View

The center view (forehead and nose) revealed variable changes across pigmented features. Mean contrast intensity decreased for brown spots (−11.86%), increased for visible spots (+11.20%), and remained relatively unchanged for red (+1.94%) and UV spots (−0.15%). Fractional area increased for all spot features, with the largest change in red spots (+170.21%). The count decreased for visible (−18.75%) and UV spots (−10.75%) but increased for brown (+14.75%) and red spots (+83.33%).

At 3 months, fractional area decreased for texture (−32.67%), pores (−39.97%), and porphyrins (−47.15%). Counts mirrored these decreases for all topographical skin features. Mean wrinkle thickness increased (+36.33%), whereas wrinkle count and total area decreased by −52.38% and −25.00%, respectively.

## Discussion

Previous generations of the VISIA-CR imaging system have been widely used in aesthetic research to evaluate the effects of laser and energy-based treatments for various conditions such as active acne, acne scars, actinic keratosis, erythema, and photoaging.^12-19^ These earlier systems typically relied on a single score per feature to follow over time, which simplified data collection but limited more nuanced interpretation. This approach also posed challenges for long-term follow-up, particularly at 6 months and beyond, when UV exposure may fluctuate greatly and significantly alter skin tone, thus confounding results.

The updated VISIA-CR 5 addresses these limitations by generating multiple analyzable parameters for each feature, enabling a more granular assessment of treatment efficacy and skin changes over time. For example, in this highlighted case, mean intensity values for pigmented spot-related features decreased at 3 months compared with baseline (with lower values corresponding to darker features), which could initially suggest worsening pigmentation when assessed alone. However, when combined with background intensity to calculate mean contrast intensity, the data actually showed an overall evening of skin tone, a clinically favorable and expected outcome following this skin rejuvenation treatment. This illustrates the value of having multiple, individual metrics within broader parameters that can offer a more comprehensive and accurate assessment, particularly in contexts where environmental variation such as UV exposure may influence pigmentation.

Similarly, an isolated increase in spot count might be misinterpreted as a negative outcome. Yet, when analyzed alongside other parameters such as fractional area, the data showed that visible and UV spots decreased in size on both oblique views, suggesting the treatment likely fragmented the original spots into smaller components rather than representing new spot formation. Contextualizing these results within the treatment this patient received, this appears to be consistent with previous literature describing the formation of numerous microcrusts filled with melanosomes following IPL treatment on pigmented lesions.^20^ Furthermore, the ability to evaluate wrinkle area within specific size categories provides insight into whether changes preferentially affect fine vs coarse wrinkles, a distinction that was previously not possible with single-score assessments. Collectively, the expanded analytical framework of the VISIA-CR 5 enhances the interpretability of skin changes and may help reduce false interpretations.

Beyond its analytical depth, the VISIA-CR 5 offers several operational advantages that enhance its utility in clinical research. The system can capture 7 images in ∼1 min, provide built-in analysis without the need for a separate application, and download data in 6 s, helping to streamline workflow without compromising quality. Additionally, it allows manual selection of regions of interest, which can be saved and consistently tracked across subsequent visits. These capabilities, combined with standardized lighting and positioning, enhance reproducibility and make the device particularly well suited for longitudinal studies assessing facial rejuvenation. This aligns with the growing trend in literature toward incorporating objective metrics alongside traditional subjective evaluation to provide a more exhaustive evaluation of aesthetic treatment outcomes.^21^

This study has several limitations. First, the case review serves primarily to illustrate the data outputs and applications of the VISIA-CR 5 system rather than to evaluate the outcomes and efficacy of the combined treatment itself, and the single patient inherently limits generalizability. Second, the relatively short 3-month follow-up may not capture longer-term skin remodeling or pigment stabilization; however, this interval was selected because of repeat procedures commonly considered around this timeframe in clinical practice. Third, although fragmentation of pigmented spots is hypothesized based on known effects of the laser and light-based treatments performed, this mechanism cannot be definitively confirmed to exclude the possibility of new spot formation in the absence of additional imaging. Fourth, the data outputs were not validated against established assessment metrics such as blinded reviewer ratings or standardized scales, other imaging systems, or previous VISIA generations. As a result, although the VISIA-CR 5 provides robust quantitative data, the clinical significance of subtle changes detected by the system cannot yet be fully established. Future studies should therefore incorporate parallel assessments, including clinician ratings, patient-reported outcomes, and direct comparison with other imaging platforms, across diverse skin types and treatment modalities. Finally, although the VISIA-CR 5 includes video capture capabilities for dynamic expression analysis, this was not evaluated in this study and represents an additional area for future exploration.

## CONCLUSIONS

This preliminary report demonstrates that the VISIA-CR 5 can provide objective, multidimensional data that can be used for individual and joint analysis to assess global skin health. Its expanded parameters offer clinically relevant insights into how features change over time, which can be particularly valuable during periods when patients may receive varying levels of UV exposure. Overall, the VISIA-CR 5 is an innovative, noninvasive imaging modality that can be used in aesthetic medicine to monitor the progress and outcomes of the skin's response to laser and other energy-based treatments.
